# Review of the Use of Digital Therapeutics for Obesity Treatment: Toward a Psychological Phenotyping

**DOI:** 10.1016/j.advnut.2026.100613

**Published:** 2026-03-03

**Authors:** Cédric Plessis, Marie Galmiche, Pierre Déchelotte, Anthony Haro

**Affiliations:** 1 PSITEC Psychologie: Interactions, Temps, Emotions, Cognition, Univ Lille, Lille, France; 2 ADEN UMR1073 “Nutrition, Inflammation and Microbiota Gut Brain Axis,” Univ Rouen Normandie, INSERM, Normandie Univ, Rouen, France; 3 Department of Nutrition, CHU Rouen, Rouen, France; 4 UFR Médecine, CHU Lille, Univ Lille, Lille, France

**Keywords:** obesity, digital therapeutics, eHealth, narrative review, psychological phenotyping

## Abstract

Digital therapeutics (DTx) offer an innovative opportunity for the prevention and treatment of obesity. However, their efficacy remains limited by a standardized approach that often does not adequately consider interindividual differences in eating behavior, motivation, mental comorbidities, and emotional regulation. Currently, many DTx rely primarily on physical indicators such as body mass index (BMI) and blood pressure, overlooking psychological factors that influence adherence and intervention success. Given that obesity is a multifactorial pathology, it is crucial to develop a more personalized approach, including psychological phenotyping. This method involves the categorization of patients according to key psychological dimensions: personality, eating behavior, psychological burden, emotional regulation, motivation, health literacy, body image, and social support. Such classification may enable optimization of DTx relevance and clinical impact, contingent on algorithm validation and integration with clinical oversight. This narrative review explores the psychological dimensions involved in obesity and how they may be used to inform personalization strategies in DTx. Through an analysis of existing studies, we identify underexplored psychological dimensions and suggest the integration in DTx to enhance patient assessment and monitoring. Finally, we discuss the clinical and methodological implications of psychological phenotyping, highlighting its value for healthcare professionals, who could achieve greater efficiency through better-tailored recommendations and optimizing patient care.

**Statements of significance**

Current digital therapeutics for obesity largely overlook psychological heterogeneity among patients, relying primarily on physiological indicators. This review is the first to propose psychological phenotyping as a structured framework, identifying 8 key psychological dimensions and 5 DTx-specific personalization mechanisms to improve engagement and clinical outcomes.

## Introduction

Digital therapeutics (DTx) are medical therapies provided in the form of applications and programs available on computers, tablets, or smartphones. DTx comprise therapeutic interventions grounded in scientific research to prevent, manage, or treat medical disorders or diseases. They can encompass various healthcare fields, such as diabetes, cancer, psychiatric disorders (depression, anxiety, addiction), and obesity [1]. They are generally prescribed alongside pharmacological treatment and may incorporate remote monitoring to facilitate patient care [2].

One of the most prevalent chronic diseases is obesity [3]. According to the WHO, obesity is a complex chronic disease characterized by excessive adipose tissue that can negatively impact health [3]. In 2022, 1 in 8 people worldwide had obesity, and this figure is continually rising [3]. In France, half the population is overweight, with 17% classified as obese [4,5]. Obesity is commonly assessed through BMI (>30 kg/m^2^), and it is a multifactorial disease involving biological, psychological, genetic, epigenetic, lifestyle, and life-history factors [6]. Obesity can lead to numerous health complications, such as type 2 diabetes, cardiovascular diseases, hypertension, cancer, sleep apnea, and depression [7]. Current treatments are based on lifestyle changes (diet, physical activity, sleep…), pharmacological therapy, or bariatric surgery, depending on the efficacy of the different interventions and the severity of the disease and its associated comorbidities.

Numerous scientific studies have investigated the multifactorial etiology of obesity, and more recently, an increasing number of studies have established a link between mental health and risk of obesity [8]. In this way, it is important to take all the variables into account when treating obesity, not forgetting the psychological dimension [9]. To achieve this, it is essential to carry out precise phenotyping of people with obesity using various methods, such as validated questionnaires. DTx may offer scalable support for mental health screening and symptom monitoring, though diagnostic accuracy and clinical utility require validation against gold-standard clinical assessments.

In this context, it is useful to distinguish between evidence-based psychological interventions delivered in digital formats and DTx as a specific class of medical interventions. Unlike the former, DTx are defined by their regulatory framework, clinical validation, and their capacity to support personalized and adaptive interventions within healthcare pathways. In fact, to be classified as DTx, the solutions must be based on scientific evidence validated by regulatory authorities, meaning they must demonstrate a direct impact on the condition of a disease without fully replacing conventional medical treatment. This enables *Conformité Européenne—*European Conformity marking and certification as medical device. It is therefore crucial to increase the body of validated scientific research to provide the medical community with robust applications supported by verified clinical and theoretical foundations [10]. DTx may offer therapeutic support to a wide population, but must adhere to these stringent criteria. Meeting such standards will allow DTx to effectively impact patients’ lifestyle changes, while simultaneously reducing costs for both patients and society [11]. Two years ago in France, the *Prise En Charge Anticipée Numérique* [(PECAN)—Early Digital Care] system was introduced to accelerate patient access to digital medical innovations, allowing early coverage by the national health insurance [3].

In the context of obesity, DTx can provide support for evidence-based interventions such as cognitive behavior therapy (CBT) or mindfulness-based cognitive therapy [9] that have been proven to be effective in the management of obesity [12]. For example, studies conducted in various countries, notably the United States (Noom) [9], Germany (Zanadio) [13], and the United Kingdom (DTxO) [14], have demonstrated the effectiveness of DTx through randomized controlled trials (RCTs). These studies have shown that applications integrating CBT-based approaches or coaching—particularly focusing on motivational aspects—led to significant weight reductions, ranging from 1.5% to 3.8% [15,16]. In a systematic review evaluating the efficacy of DTx interventions, Kocol et al. [17] suggest combining CBT approaches with rigorous psychoeducation to reduce depressive symptoms commonly associated with obesity. In this way, DTx could enable early psychological phenotyping and management of patients with obesity to offer them the therapeutic program best suited to their needs. DTx could therefore help screen for obesity and various associated comorbidities in primary care because individuals with obesity often experience their first therapeutic encounter with a general practitioner [18]. These examples illustrate how DTx platforms can support personalization and delivery beyond traditional in-person interventions. However, in clinical practice, the limited duration of consultations generally does not permit a full examination of patients' clinical situation.

However, current DTx present several limitations, notably because they frequently offer standardized solutions that do not sufficiently consider users' individual characteristics. Real-time measurement of multifactorial variables, such as behaviors, emotions, cognition, and motivation, would allow interventions to be adapted in a more personalized and effective manner [9]. Nevertheless, the major challenge for DTx is user engagement. Indeed, long-term user engagement is essential for ensuring the efficacy of DTx, which frequently experience high attrition rates [19]. Personalization is also an increasingly important criterion to ensure user engagement in the era of precision medicine [20]. Treatment adherence improves when patients feel personally understood [20], especially through tailored communication [21].

This narrative review explores which are the psychological dimensions involved in obesity and how they may be used to inform personalization strategies in digital therapeutics (DTx), with a focus on their clinical relevance for engagement, adherence, and long-term effectiveness. Based on existing evidence, the review outlines a psychological phenotyping framework to support personalized DTx interventions in obesity [22].

**FIGURE 1:**
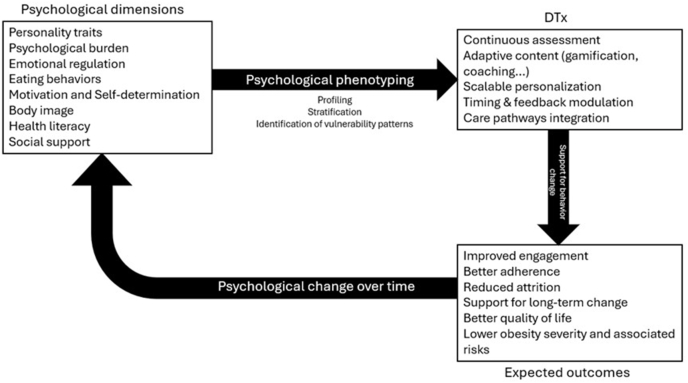
Conceptual framework of links between psychological dimensions, psychological phenotyping, and personalization mechanisms in digital therapeutics for obesity.

A conceptual framework summarizing the scope of this review and the links between psychological dimensions, phenotyping, and DTx personalization is presented in Figure 1.

## Methods

Narrative reviews allow for an informal synthesis of knowledge on a topic by offering a comprehensive discussion. Here, we propose the concept of psychological phenotyping to enhance personalization in DTx to optimize prevention and support for people suffering from obesity. This narrative review discusses existing knowledge and serves as a foundation for future research on this topic. It is an effective format for presenting knowledge about a specific subject [23].

To construct this narrative review, we followed a methodology inspired by recommendations from Saracci et al. [24]. The primary objective of this article is to synthesize current knowledge on psychological phenotyping in the context of obesity and to examine its integration into DTx to improve the personalization of digital interventions and optimize prevention and care. We conducted a literature search primarily using the PubMed and Cochrane Library databases, selected for their relevance to medical and psychological fields. The selected period ranged from 2003 to 2024, as 2003 marks the official recognition of obesity as a global epidemic by the WHO [3]. Given the rapid evolution of digital health technologies, particular attention was paid to including recent studies (within the past 5 y) to capture the most current innovations in DTx.

The following keywords were used in various precise combinations: “Obesity,” “Overweight,” “Psychological,” “Mental Health,” “Digital Therapeutics,” “eHealth,” “Digital Therapy,” “Phenotype,” and “Cognitive Behavioral Therapy.” Searches were conducted exclusively in English to ensure homogeneity of the results.

Inclusion criteria consisted of:

• Randomized controlled trials, systematic reviews, meta-analyses, or rigorous observational studies published in peer-reviewed journals;
• Studies specifically focusing on digital interventions for treating or preventing obesity or overweight in adults (BMI ≥ 25);
• Studies explicitly incorporating psychological variables (e.g., motivation, emotional regulation, treatment adherence…);
• Given the regulatory focus of the review on DTx as a specific class of medical devices, particular attention was paid to interventions meeting DTx criteria.

Exclusion criteria were as follows:

• Non-peer-reviewed articles, pilot studies with small sample sizes (<10 participants), opinion pieces, or editorials;
• Digital interventions lacking an explicit psychological component or without direct relevance to obesity management.

The study selection process occurred in 2 stages: an initial screening based on titles and abstracts, followed by an in-depth analysis of full-text articles.

Extracted information was organized into summary tables to facilitate comparative analyses across the following dimensions: clinical effectiveness, characteristics of proposed psychological phenotyping, personalization strategies, and practical implications for the use of DTx.

Finally, we established an operational definition of psychological phenotyping in the context of obesity to facilitate the interpretation of results and provide practical recommendations for future personalization of digital therapeutics.

## Results and discussion

The psychological dimensions identified include personality, psychological burden, eating behaviors, emotion regulation, motivation, personal and social body image, social support, quality of life, and health literacy. We provide a brief description of these dimensions based on existing scientific literature, followed by essential personalization elements for DTx support. These results are also summarized in Table 1.

**TABLE 1:** Psychological determinants of obesity and their relevance for digital therapeutics personalization

Psychological factor	Key elements	DTx features
Personality	Neuroticism	High: Empathetic coaching and stress-management Low: Challenges and direct feedback if low
	Conscientiousness	High: Structured interfaces, planning features Low: Frequent coaching prompts and simplified goal setting if low
	Openness to experiences	High: Gamification needed for stimulation if high levels Low: Predictable routines, stable interface
	Extraversion and agreeableness	High: Social features or motivational messages Low: Self-paces modules, independent pathways
Psychological burden	Depression	Symptoms monitoring, combined with professional care in severe cases
	Bipolar disorder	Emotional regulation features, mood tracking, stabilization-focused content
	Stress	Coping support tools, stress threshold monitoring
	Ruminations	Cognitive support strategies, mindfulness and CBT tools (thought records)
Eating behaviors	Eating disorders	CBT tools, mindfulness eating, real-time monitoring of thoughts, emotions, and eating habits
	Emotional eating	Emotion-triggered just-in-time prompts, emotion journaling, mindful eating
	Food addiction	Craving management, relapse prevention tools, trigger identification planning
General functioning	Emotional intelligence	Emotion recognition and management training, alexithymia-adapted content
	Self-determination	Personalized content to enhance autonomy, competence and relatedness
	Extrinsic motivation	Transformation into more internalized forms, social connections and coaching help
	Quality of life	Self-compassion and motivational content
	Self-efficacy	Support with feedback and realistic goals, motivation interviewing tools, and graded goal difficulty
	Body dissatisfaction	Introduce self-affirmation, mirror exposure, and self-compassion exercises
Body image	Body dysmorphic disorder	Sensitive design to avoid reinforcing a negative image
	Stigma and cultural norms	Challenge stereotypes, providing inclusive and neutral representations, stigma-reduction psychoeducation
	Media and peer influence	Incorporate media literacy and peer support content
	Self-esteem	Positive feedbacks and self-compassion
	Parental influence	Positive modeling without pressure, guide for family-based support
	Fragmented information	Provide continuous and structured knowledge flow
Health literacy	Nutrition	Adapt content to literacy level, multimodal delivery (text, images, videos)
	Healthcare professionals	Align with medical advices, correct misinformation
	Food insecurity	Address emotional and nutritional impact, propose solutions within DTx
	Online health communities	Integrate peer discussion and emotional sharing spaces
Perceived social support	Connections	Build perceived support even with limited connections

DTx, digital therapeutics.

### Psychological dimensions of obesity

#### Personality

Within the framework of the 5-factor personality model (Big Five), a systematic review indicated that high neuroticism, characterized by a focus on negative events, could be, through impulsivity and sensation-seeking behaviors, a risk factor for mental health issues and associated behaviors, including obesity [25]. For example, 1 study reported in a large Italian population that impulsivity is a key link between neuroticism and obesity [26]. Similarly, Kakizaki et al. [27], examining a cohort of >50,000 individuals in Japan, identified an association between neuroticism and low agreeableness, reinforcing the notion that personality profiles significantly influence individual behaviors. On the other hand, conscientiousness, via self-control, could act as a protective factor [28]. One study also showed that subjects with obesity (BMI ≥ 35) had significantly higher novelty seeking than lean subjects (BMI < 25) (*P* < 0.001) [29].

The impact of neuroticism on the risk of obesity can be partially explained by the positive correlation between neuroticism and emotional eating, namely the tendency to engage food intake to regulate emotions. These findings are corroborated by several studies, as summarized in a meta-analysis [25].

Nevertheless, there are some studies with contradictory results, which could be justified by the methodology used and the population studied with both sociocultural and phenotypic differences. In fact, a meta-analysis of population-based studies showed a correlation between neuroticism and obesity in European and Australian populations, but not in American populations [30]. Bangjuk et al. [29], for instance, found that longitudinal studies identified an association between obesity and extraversion and agreeableness, but not neuroticism, the latter being primarily identified in cross-sectional studies. Such differences may be explained by the questionnaire used, as well as sociodemographic differences between the studied cohorts (i.e., agreeableness increasing with age, differences in education level).

Personality could also have an impact on the success of care. People who successfully lose weight through these programs usually exhibit lower novelty seeking, possibly because high novelty seeking might be associated with boredom susceptibility, which negatively impacts engagement in care and perseverance but also contributes to increased food intake. According to the same study, individuals with obesity often display weaker self-control, a finding consistently documented in the literature [31].

#### Psychological burden

##### Self-esteem

Obesity is associated with several psychological comorbidities, including low self-esteem, anxiety, and depressive symptoms, although these links are not always clear [32]. All these factors contribute to a reduced quality of life for these individuals.

Regarding self-esteem, the relationship with obesity is bidirectional: weight loss can enhance self-esteem, but participating in a weight-loss program can conversely weaken it, especially if participants fail to achieve their set goals [33]. This process may also modify pre-existing coping strategies.

##### Depression

The relationship between obesity and depression is also bidirectional: depression can lead to weight gain, and obesity can, in turn, exacerbate depressive symptoms. These effects are particularly pronounced among individuals with a BMI exceeding 40 (morbid obesity). However, these links are weaker in cases of non-clinical obesity [34]. Depression could also impact the perception of body image. Although the directionality between depression and body image remains complex, evidence suggests that childhood depression can significantly increase the risk of adult obesity, partly through its long-term effects on social and cognitive functioning [35]. Furthermore, negative self-image tends to be more prominent when obesity begins at an early age. Finally, some studies suggest that certain antidepressants, particularly selective serotonin reuptake inhibitors, may contribute to weight gain over the long term by increasing appetite and slowing metabolism [36].

##### Stress and anxiety

Stress and anxiety are now recognized as key contributors to both the development and persistence of obesity, primarily through the activation of the hypothalamic-pituitary-adrenal axis, elevated cortisol levels, and increased systemic inflammation [37]. They can also modulate the composition of the gut microbiota, leading to dysbiosis. This disruption is associated with metabolic and inflammatory imbalances, representing a risk factor for obesity. Conversely, dysbiosis can also affect the regulation of the hypothalamic-pituitary-adrenal axis, creating a bidirectional microbiota-gut–brain axis that contributes to the modulation of energy metabolism. In addition, stress and anxiety promote emotional eating, which, as described in the following paragraph, is itself a significant risk factor for obesity [38]. There can be many sources of stress, which include relationships with parents, professional status, promotion of health behaviors within the family, or childhood trauma.

##### Quality of life

Overall, individuals with obesity experience a lower quality of life, marked by increased discrimination, reduced access to higher education, fewer marriage opportunities, and wage disparities, particularly among females. Workplace discrimination becomes especially pronounced at a BMI of 35 and above. Among adolescents, obesity is associated with lower academic persistence, higher absenteeism, and reduced educational attainment, with a stronger impact observed in females [35]. Obesity is also associated with lower self-esteem, which may mediate or exacerbate the psychological distress and social withdrawal experienced by affected individuals [39]. Additionally, individuals with obesity often face multiple comorbidities, which can further compromise quality of life. Functional limitations, including difficulties in mobility and performing daily activities, also contribute to the burden experienced by individuals living with obesity [40].

##### Other mental health

Obesity is also associated with other mental disorders, such as schizophrenia and bipolar disorder, although these relationships would not be direct [41]. In fact, bipolar disorder is a frequent comorbidity in obesity, and the co-occurrence of binge-eating disorder (BED) and bipolar disorder in individuals with obesity may lead to more severe eating disorder symptoms and mood disturbances, such as depression [42].

#### Eating behavior and eating disorders

There is a strong overlap between obesity and eating disorders, regardless of the form (i.e., anorexia, bulimia, or BED)—highlighting the complex and bidirectional relationship between weight status and disordered eating behaviors [43]. Obesity affects 28.8% of individuals with an eating disorder, currently or has been previously reported. Unsurprisingly, BED is the form of eating disorder most strongly associated with the onset of obesity [44].

Different dimensions of eating behavior could be measured by the Dutch Eating Behavior Questionnaire, such as emotional eating or external eating [45]. A recent study reported that 58% individuals with obesity exhibit emotional eating, which is particularly prevalent among females (65.9%) [46], especially those experiencing depression. Furthermore, emotional eating scores were significantly higher in patients with obesity and BED compared with individuals with obesity without BED [47]. Emotional eating is defined as the tendency to eat in response to emotions. Although it is not considered an eating disorder, emotional eating constitutes a behavioral response influenced by emotional state. Emotional eating episodes are generally characterized by a loss of control over food intake. Moreover, food choices in such contexts are often calorically dense, highly palatable, and low in nutritional value, serving as a coping strategy for managing negative emotions and making emotional eating a significant contributor to weight gain and challenges in achieving weight loss. However, this strategy paradoxically increases negative feelings due to resulting guilt, triggering mental rumination. Moreover, emotional eating may also be influenced by depressive symptoms, also contributing to the development of obesity [32].

Furthermore, in this context, an assessment of cognitive restraint appears particularly relevant. Cognitive restraint refers to the intentional and repeated limitation of food intake aimed at controlling body weight. However, cognitive restraint is closely intertwined with emotional eating and loss of control, thereby increasing the risk of overeating, particularly promoting a feeling of frustration.

In some individuals, eating behaviors may extend beyond emotional or externally driven patterns toward compulsive or addictive-like responses to highly palatable foods, characterized by craving and loss of control. Although there is currently no consensus in scientific literature, an increasing number of studies have described food addiction-like behaviors. Food addiction is characterized by the consumption of highly palatable, energy-dense foods in the absence of physical hunger and despite negative consequences. Food addiction may represent a contributing factor to obesity and BED. Studies have reported that 25% to 37% of individuals with obesity exhibit food addiction, with prevalence reaching ≤60% in patients with class III obesity [48]. Moreover, over 90% of patients with BED also present with food addiction, and a significant correlation between BED and food addiction symptoms has been established [49,50]. The Yale Food Addiction Scale can be easily used to evaluate food addiction symptoms.

#### Emotional regulation

Rumination involves repetitive thoughts focused on concerns perceived as difficult to control. As an emotional regulation mechanism, it increases the frequency of negative emotions and can provoke loss of control, overeating due to a depletion of cognitive resources in response to emotional distress. In a vicious circle, mental rumination contributes to emotional eating. In fact, feelings of guilt after episodes of emotional eating may paradoxically fuel further rumination, reinforcing the cycle [51]. Emotional eating then becomes ritualized, turning into a low-effort habit that conserves cognitive resources [52].

Another specificity is related to emotional intelligence [53], defined as the ability to perceive and understand one’s own emotions as well as those of others. Emotional intelligence, a protective factor in various life domains, is linked to greater life satisfaction and helps avoid emotional eating responses. Individuals with obesity notably experience difficulties in emotion identification, with sometimes alexithymia, often related to the tendency to avoid negative emotions [54], or challenges with emotional intelligence tasks [55]. One study showed that people suffering from severe obesity (BMI ≥ 40) were characterized by significantly lower emotional intelligence and a greater tendency to use suppression as an emotion-regulation strategy, compared with people of normal weight [52]. fMRI studies also reveal emotional dysregulation at the brain level in patients with obesity compared to healthy controls, notably through reduced activation of the ventromedial prefrontal cortex, responsible for regulating negative emotions in interaction with other brain regions. Similar dysfunctions have also been observed in addiction studies involving substances [56].

Emotion dysregulation has also been implicated in the co-occurrence of obesity and addictive behaviors. Individuals with obesity may turn to substances such as alcohol, tobacco, or cannabis as maladaptive strategies to manage negative emotions, in parallel with emotional eating patterns. This overlap suggests shared underlying mechanisms between substance use and dysregulated eating, particularly involving impulsivity and affect-driven coping strategies [57,58].

#### Motivation

Self-determination theory posits that a context promoting personal self-determination by satisfying the 3 basic psychological needs—autonomy, feeling competence, and relatedness—leads to enhanced psychological well-being [59]. Conversely, externally regulated motivation results in fewer positive outcomes or even negative consequences for well-being [59]. Extrinsic motivation stems from external factors and can be subdivided according to the degree of internalization of the behavior: external regulation (exercising because a doctor recommends it), introjected regulation (exercising to avoid feeling guilty), identified regulation (exercising due to an understanding of its health benefits), and integrated regulation (exercising because being active aligns with one's desired identity). Intrinsic motivation is the most self-determined form of motivation, characterized by engagement driven by enjoyment and direct satisfaction from the activity itself (e.g., exercising simply because one enjoys it).

From an applied perspective, self-determination theory illustrates that motivation in healthcare programs tends to become more self-determined over time. However, a study on motivation in prescribed exercise programs suggests that self-determined motivation may be strengthened through autonomy support provided by trained supervisors, as well as the satisfaction of basic psychological needs (competence and relatedness). These mechanisms could help improve participants’ adherence to the program [60]. This shift was partly attributed to limited initial contact with program supervisors and a lack of understanding of the long-term benefits of exercise. Moreover, insufficient social support, particularly at the beginning of the program, may hinder the fulfillment of the relatedness need. Nonetheless, this barrier can sometimes be overcome by fostering new social connections within the program environment.

Other studies have emphasized the impact of self-efficacy on overweight and obesity prevention. Overweight individuals and people with obesity often report relatively high levels of self-efficacy for initiating behavioral change, especially in structured intervention settings. However, this self-efficacy may not always translate into sustained behavior over time, particularly when compared to normal-weight individuals or when facing environmental and emotional barriers [61]. However, they require support to maintain this self-belief, particularly through tailored education about the benefits of behavioral changes [62]. Promoting self-efficacy and self-determination, reinforcing basic psychological needs through clear, positive feedback, realistic goal setting, and providing individuals with choices (e.g., offering multiple options) are effective strategies.

Motivational interviewing (MI) is a commonly employed solution in weight-loss interventions. This therapeutic approach focuses on individuals’ ambivalence and aims to foster intrinsic motivation for change. MI explores individuals’ personal reasons, assisting them in autonomously setting realistic goals. This interviewing style avoids confrontation, prioritizing empathy to gradually encourage behavior change. MI is frequently applied in addiction treatment. However, a recent systematic review discussing remote MI for weight loss yielded mixed results: only half of the interventions using this approach showed significant outcomes. The most successful study involving MI was conducted via telephone. Overall, MI is found to be as effective as other therapeutic approaches in weight loss contexts [63], but its efficacy improves when combined with CBT, particularly in promoting physical activity [64].

#### Body image

##### Self-body image

Self-body image is a complex and multidimensional construct composed of cognitive schemas, affective responses, and perceptual interpretations that shape how individuals perceive, feel about, and mentally represent their own appearance [65]. This perception plays a crucial role in behavior and psychosocial functioning. Among body image-related difficulties, body dissatisfaction refers to a negative evaluation of one’s appearance, often involving a desire to modify certain features. It is widespread, particularly among females, and although distressing, it does not necessarily involve a distorted perception of the body. By contrast, body dysmorphic disorder is a more severe and clinical condition marked by an intense preoccupation with perceived physical flaws that are either minimal or nonexistent to others, involving a significantly distorted body image. These disorders are often associated with emotional distress and functional impairments.

Both body dissatisfaction and body dysmorphic disorder can lead to maladaptive behaviors, especially those related to eating habits. Individuals may skip meals, eat rapidly (tachyphagia), induce vomiting, use diet pills and laxatives, or experience episodes of emotional eating. Additionally, a negative perception of one’s body may result in compulsive behaviors such as mirror checking or avoidance, excessive exercise, or repeated requests for cosmetic procedures. These behaviors are strongly associated with decreased self-esteem, depressive symptoms, and an increased risk of developing eating disorders and obesity [66].

Risk factors for poor self-body image include the degree of overweight, being female, binge-eating behaviors, age, anxiety symptoms, depression symptoms, thin-ideal internalization, perfectionism, neuroticism, and early-onset obesity [54,67,68]. In contrast, engaging in regular physical activity may serve as a protective factor against body dissatisfaction [69]. Body image can be broken down into 4 main constructs: dissatisfaction with appearance or weight, overvaluation of weight, excessive concerns about appearance or weight, and intense fear of weight regain [70]. Notably, self-body image is more severely impaired in individuals with bulimia compared to those with BEDs, and even more so than in individuals without associated disorders.

Poor self-body image is strongly associated with various negative outcomes, including increased psychological distress, eating disorders, lower quality of life, and reduced self-esteem [71]. Although these effects can be observed across populations, they tend to be less pronounced in males. In some cases, being overweight or obese in males is culturally linked to perceptions of strength and resilience [72]. In contrast, females generally report greater body dissatisfaction, often focused on specific body parts such as thighs, hips, or abdomen. Interestingly, although females are more dissatisfied with individual features, males may report lower satisfaction with their overall appearance, partially reversing the trend at a global level [71].

However, it remains important to recognize that body image can act as a barrier to appropriate emotional regulation, particularly for individuals with a history of being overweight. Even after significant weight loss, these individuals may continue to perceive themselves negatively, sometimes referred to as "phantom fat,” which highlights the persistence of negative self-perceptions [73].

The stigma surrounding obesity is a key factor influencing body image. Body image is shaped by prevailing beauty standards, which vary across individuals, cultures, countries, and historical periods. These standards are predominantly constructed and disseminated through media channels [6,74]. In fictional media, characters with obesity are rarely depicted as leaders or involved in romantic relationships; instead, they are often portrayed as lacking control over food, frequently engaging in binge-eating behaviors. Such representation reinforces the stigmatization of individuals with obesity, who are often attributed personal responsibility for their condition (attribution theory). They are perceived as lacking willpower, being lazy, incompetent, and emotionally unstable, further reinforcing societal prejudices against them [68].

##### Social body image

Social body image is primarily shaped by how individuals perceive others' judgments, comparisons with peers, and societal pressure to conform to beauty standards imposed by media, peers, and parents.

Social media usage presents an additional threat to body image. In fact, social media have emerged as particularly influential in shaping body image perceptions. A growing body of scientific literature indicates that both sharing and viewing images on social media, as well as the number of online friends, are associated with the risk of body dissatisfaction [67,75]. Furthermore, a positive correlation between the use of social media and body dissatisfaction has been observed in both females and males, although the strength of this association tends to diminish with age [76]. This is because social media platforms provide direct and immediate opportunities for social comparison, reinforce aesthetic norms, and amplify these standards through interactions such as “likes” [77]. A key underlying mechanism underlying body dissatisfaction is the internalization of the thin ideal. This refers to the cognitive assimilation of a socially constructed standard of thinness, with psychological consequences for the individual. Empirical evidence suggests that the more strongly individuals internalize the thin ideal the more likely they are to experience body dissatisfaction with potential negative consequences for psychological well-being. In fact, the consequences of this stigma affect various aspects of life, including education, employment, healthcare, and significantly, psychological well-being and self-esteem [78]. Video-based social media platforms, particularly those dominated by influencers, appear to be the most problematic in this regard.

Peers can act as a protective factor. When an overweight individual is part of a social group composed predominantly of overweight individuals, they experience less social comparison pressure, which mitigates the negative impact on their body image [79]. Additionally, engagement in social relationships has been shown to act as a protective factor for body image [80]. Parents also play an important role: promoting healthy eating habits and regular physical activity leads to positive effects on BMI. However, this is only effective in the absence of parental pressure [80].

Furthermore, the media play a crucial role in reinforcing beauty standards. By encouraging constant comparison between the idealized body image portrayed and one's perceived self-image, they contribute to multiple effects: psychological (deterioration of body image), motivational (pressure to conform to thinness and/or muscularity ideals), and behavioral (excessive exercise practices or the development of eating disorders). Research indicates that the greater the discrepancy between one's ideal body and perceived self-image, the higher the level of body dissatisfaction, particularly among females [81].

#### Social support

Therapeutic compliance is essential to ensure treatment effectiveness, particularly in chronic diseases. However, only 50% of patients follow their prescriptions correctly [82]. This lack of compliance puts their health at risk, often due to forgetfulness or low engagement. Conversely, high adherence to treatment extends beyond its direct effects on the disease: it also strengthens self-efficacy and patient empowerment, improving interactions with healthcare professionals [83]. This empowerment translates into more active participation in medical consultations and better day-to-day disease management [84]. Furthermore, the effects of social support are not limited to medical contexts; they also influence weight management behaviors. For instance, individuals with obesity surrounded by close acquaintances aiming to lose weight are 4 times more likely to adopt the same goals [85].

Social support, whether direct or indirect, plays a crucial role in improving compliance. Online Health Communities (OHCs) provide an anonymous space where patients can share their experiences, symptoms, and emotions. These interactions offer both social and emotional support, which, over time, fosters greater engagement with treatments [86]. However, the effectiveness of OHCs partly depends on health literacy, which is patients' ability to understand and use the medical information shared within these communities [84]. Additionally, the perception of social support, rather than its actual presence, plays a key role. Even in the absence of deep friendships or family connections, the simple feeling of being supported can yield positive effects. This explains the growing interest in OHCs, as they help participants feel part of a group with shared concerns. Nevertheless, not all OHCs are equally reliable, and ensuring that they disseminate accurate health information is crucial to prevent counterproductive effects [87].

Beyond compliance and eating behaviors, stress experienced during childhood or adolescence is a significant risk factor for obesity in adulthood. This link is explained by elevated cortisol levels, lack of physical activity, sleep disturbances, and an increased preference for high-calorie foods. However, a supportive social environment can mitigate these effects by fostering a sense of security, boosting self-esteem, and reducing the impact of stress. Studies indicate that the absence of interpersonal stress during early life is directly associated with lower BMI in adulthood, regardless of other stressors such as financial difficulties [88].

Finally, social support varies depending on its source. Structural support refers to social integration and the availability of close relationships, whereas functional support is defined by the perceived quality of received support, which depends on individual factors such as personality or emotional state. Despite the observed benefits, researchers remain cautious about the effects of social support, particularly regarding interactions with other obesity-related factors (e.g., social support linked to smoking behaviors) [76].

#### Health literacy

Health literacy encompasses the social and cognitive skills required to understand and use information to promote and maintain good health (WHO). It plays a crucial role in obesity management. Low health literacy is associated with significant consequences, such as increased hospitalizations, higher reliance on emergency services, poor treatment adherence, and more frequent use of medical care—effects that are even more pronounced among older adults [89].

In obesity management, nutrition constitutes a fundamental pillar alongside physical activity and CBT. However, the effectiveness of interventions depends not only on access to nutritional knowledge but also on the skills necessary to integrate this knowledge into daily life, aligning with the broader concept of health literacy. Studies show that when education is limited to mere knowledge transfer without guidance on practical skills, weight regain is commonly observed. Despite the abundance of educational resources, individual literacy levels are rarely assessed, which may limit the effectiveness of educational approaches [90].

Another key challenge is the fragmentation of nutritional information (e.g., through mass media or social networks), which hinders sustainable behavioral change. Research suggests that fostering healthy eating habits requires the continuous and dense provision of knowledge. Conversely, fragmented information has little impact on obesity outcomes [91].

Health literacy is also crucial among healthcare professionals, as it directly influences the quality of patient care. However, many stereotypes about individuals with obesity persist within the medical community. For example, 60% of nurses obtain their information about bariatric surgery from mass media, whereas only 5.6% acquire it through formal training. Nearly one-third of healthcare professionals lack adequate knowledge about obesity. Although BMI-related concepts are generally well understood, knowledge gaps are more prevalent concerning bariatric surgery (51.1% correct responses) and recent pharmacological treatments (38.6%). However, although healthcare professionals often perceive their knowledge as insufficient, objective assessments indicate that their actual knowledge is generally better than their self-perceptions suggest [92].

Beyond knowledge and literacy, food insecurity, the fear of having limited access to quality food necessary for a healthy life, often due to financial difficulties, is a pressing issue. It is strongly associated with high-risk behaviors, anxiety and depressive disorders, and obesity itself, particularly among females. Food insecurity thus represents both a social and nutritional problem that requires comprehensive solutions, with health literacy playing a key role [93].

### Tailoring DTx for obesity

Translating psychological phenotyping into actionable DTx features requires specific technical architectures that distinguish regulated therapeutic software from general wellness applications. We identify 5 core mechanisms through which DTx platforms may operationalize phenotype-informed personalization:

1. Personalized module assignment: Rule-based algorithms assign intervention modules (e.g., CBT skills training, meal planning, urge surfing techniques) based on baseline phenotype assessment and ongoing reassessment data. Implementation requires predefined decision trees ensuring clinical logic transparency and allowing clinician review of assignment rules.
2. Just-in-time adaptive interventions: Context-triggered micro-interventions deliver brief skills (e.g., 3-min mindful breathing prompts when self-reported craving intensity >7/10; social support reminders before identified high-risk times) based on ecological momentary assessment or passive sensing data (location, time of day, app usage patterns, and daily habits). This approach targets moments of highest vulnerability while minimizing user burden, though it requires validation of trigger algorithms and assessment of whether real-time interruptions enhance or disrupt coping.
3. Stepped-care escalation: Pre-specified symptom thresholds trigger automated alerts to supervise clinicians or platform-initiated check-ins to ensure safety monitoring while preserving autonomous engagement for stable users. Examples include: PHQ-9 ≥15 (moderate-severe depression), digitally sustained disengagement, or rapid weight changes (5% body weight loss or gain). Implementation requires clear protocols for clinician response times, emergency referral pathways, and fail-safe mechanisms when automated systems malfunction.
4. Engagement-based tailoring: Dynamic adjustment of coaching frequency, gamification density, and feedback tone based on usage patterns. For example: reducing notification frequency for high-engagement users to avoid saturation effects; increasing human touchpoints (clinician messages, peer support prompts) for users showing early disengagement signals; modifying goal difficulty after consecutive non-completion to prevent learned helplessness. Effectiveness depends on distinguishing disengagement from appropriate self-regulation (the user achieving independence) compared with dropout risk.
5. Hybrid human-AI decision support: Algorithm-generated recommendations (e.g., suggested goal modifications, module switches, intensity adjustments) presented to supervising clinicians for validation before implementation. This approach maintains accountability and clinical judgment while leveraging computational pattern detection across large user datasets. Examples include AI flagging users with depression, high neuroticism, and low social support as high-risk for dropout, with a clinician deciding whether to intensify outreach or refer to in-person care. These mechanisms require validation through micro-randomized trials (testing specific personalization rules), algorithm audits for bias (ensuring equitable performance across demographic subgroups), and longitudinal studies assessing sustained effectiveness beyond initial engagement. Critically, transparency in decision rules, clinician override capacity, and user understanding of how their data inform personalization remain essential to maintain safety standards, regulatory compliance, and informed consent.

In the following sections, each psychological dimension is discussed with respect to its relevance for DTx personalization and how it may be operationalized within digital therapeutic frameworks.

#### Personality

Personality traits are particularly relevant for DTx because their relative stability allows early assessment and sustained personalization through adaptive, data-driven intervention delivery. As novelty seeking is frequently associated with obesity, DTx, to increase their effectiveness, should regularly stimulate users by incorporating gamification elements [94]. Conscientious individuals may prefer a minimalist, structured interface offering detailed monitoring features and rigorous planning [95]. Conversely, frequent coaching should be provided for users with lower conscientiousness [96]. Although chatbots are frequently used in DTx [97], it would be interesting to adapt the chatbot's speech to the personality of the participant using artificial intelligence. For example, individuals with low extraversion and agreeableness may benefit from self-coaching or independent engagement with content. Conversely, those more socially oriented may prefer support groups or personalized motivational messages. The content and feedback given by the DTx could also be adapted to the personality. Users with low neuroticism typically manage challenges and stress well [98] and may benefit from direct feedback. In contrast, users high in neuroticism will likely prefer empathetic coaching and stress-management techniques, with regular adaptations to encourage adherence.

From a DTx perspective, personality phenotyping can be operationalized through adaptive coaching style, defined by tone, feedback frequency, goal granularity, and reinforcement schedules. Implementation requires transparent decision rules with clinician override capacity to maintain safety and accountability, particularly when personality traits intersect with clinical risk factors (e.g., high neuroticism with elevated depressive symptoms).

#### Psychological burden

In the context of DTx, psychological comorbidities are particularly relevant because they can be continuously monitored and integrated into adaptive care pathways, allowing DTx to adjust intervention intensity while remaining coordinated with clinical follow-up when needed. These digital tools can be connected to remote monitoring systems [99] that track psychiatric symptoms (i.e., depressed mood, anxiety, loss of energy, sleep disturbances, brain fog) as well as metabolic and cardiovascular parameters (blood glucose, blood pressure, heart rate, sleep). Personalizing DTx for obesity treatment could incorporate these elements to construct an effective therapeutic experience. However, in the case of severe forms and significant associated comorbidities such as major depression, their efficacy is limited; that is why they need to be accompanied by care from a healthcare professional. In fact, in cases of major depression, preliminary evidence suggests that DTx-delivered interventions show modest effect sizes compared to treatment-as-usual controls, though these findings remain limited to supervised care settings and do not replace specialist psychiatric management [100].

Accordingly, DTx should not replace clinical management of psychiatric comorbidities but can support it through symptom monitoring (e.g., weekly PHQ-9 for depression, GAD-7 for anxiety, scales for mood tracking) and predefined escalation pathways to a healthcare professional when risk thresholds are reached. These implementations require validated cutoff scores, clear protocols for emergency referral, and clinician information with defined response time expectations. For severe comorbidities, DTx serve as adjunctive tools within integrated care models rather than standalone interventions.

#### Eating behaviors

CBTs have emerged as a cornerstone in the care of the emergency department (ED). By targeting maladaptive thoughts, beliefs, or emotional responses and their association with food intake, CBT helps individuals identify triggers for emotional eating, binge episodes, or food addiction-like behaviors and develop healthier coping strategies.

DTx platforms have begun to incorporate CBT-based modules for ED, though evidence remains primarily at the feasibility stage. They can deliver CBT-based interventions while overcoming common limitations of traditional therapy, such as accessibility barriers, interruptions between sessions, and a lack of flexibility. By extending therapy beyond the clinic, DTx provides continuous support and reinforcement of behavioral strategies. Real-time monitoring of thoughts, emotions, and eating habits further enables patients to gain insight into their eating behaviors, facilitating the implementation of personalized, evidence-based coping strategies. However, DTx-specific evidence for ED treatment in obesity populations remains limited and requires further validation through RCTs.

DTx can translate eating-behavior phenotypes into personalization by combining repeated in-the-moment assessments with just-in-time prompts and adaptive module assignment (e.g., stimulus control, meal planning, urge surfing) based on identified triggers. Users may complete pre- and post- meal ecological momentary assessments capturing hunger levels, emotional states, eating context, and post-meal satisfaction or guilt. These data inform personalized interventions: users demonstrating high external eating patterns receive stimulus control models including environmental restructuring guidance, strategic grocery shopping controls, and cue exposure with response prevention exercises. Users with elevated emotional eating access emotion-regulation skill modules including urge surfing, opposite actions, and emotional differentiation training.

#### Emotional regulation

To optimize DTx, regular and effective measurement of emotions should be considered. This could involve keeping an emotional journal and being associated with some strategies to reduce the risk of emotional eating. Relaxation methods (considering the previously described personality traits) or techniques to interrupt emotional eating —such as mindful eating—have shown efficacy in traditional face-to-face settings [98] and represent promising candidates for DTx adaptation, pending validation of digital delivery formats through controlled trials comparing app-delivered with therapist-delivered mindfulness protocols. Automatic thoughts resulting from maladaptive cognitive schemas or low emotional intelligence can be challenged using simple CBT tools (e.g., Beck’s thought records) to identify more adaptive alternative thoughts. These tools facilitate transitioning from maladaptive emotion-focused coping strategies toward more problem-focused solutions [101].

Within DTx, emotion-regulation profiles can be leveraged via brief, timely skills delivery (CBT-informed techniques) triggered by self-reported affect ratings, craving intensity, or patterns of disengagement, thus targeting moments of highest vulnerability. For users reporting high negative effects, automated prompts deliver guided skills including breathing exercises, progressive muscle relaxation files, or brief mindfulness practices, drawing on CBT interventions. For people demonstrating alexithymia patterns, psychoeducation modules include emotion differentiation exercises, body sensation mapping, and emotional granularity training to build interoceptive awareness.

#### Motivation

In DTx, motivation represents a fundamental pillar [102]. Assessing a user’s motivational state at specific times and repeatedly over time can enable adaptive personalization of application tools to facilitate the internalization of behavior change and sustain long-term engagement. Gamification can stimulate intrinsic motivation through engaging challenges, whereas compassionate and encouraging messages can minimize externally regulated behaviors. Understanding a user's values can foster identified motivation, positively influencing engagement and psychological well-being. Moreover, the use of chatbots for MI remains at an early stage but appears promising [103]. Although conversational agents can operationalize MI (open-ended questions, reflective listening, autonomy support), their integration into regulated DTx requires a validation of therapeutic fidelity, safety protocols for detecting ambivalence about self-harm, and non-inferiority trials comparing chatbots with human-delivered interviewing. Elements such as open-ended questions, emotional validation, subjective motivation scales, progress visualization, highlighting patient dilemmas, or strategies to transform doubts into actions are components capable of promoting user change [104]. Such applications can maintain continuity of care provided by healthcare professionals, who are often regularly trained in MI [105].

DTx can personalize motivation strategies by dynamically adjusting goal difficulty, feedback type, and MI consistent messaging based on engagement signals and user preferences, while minimizing dropout through early detection of disengagement. Specific personalization includes goal-adjustment difficulty with dynamic adjustments to prevent learned helplessness after non-completion, feedback type adaptation with low self-efficacy people receiving praise emphasizing effort and strategy use rather than outcome-focused praise, values-aligned messaging, early disengagement detection, and operationalizing MI principles in chatbots.

#### Body image

In the context of DTx, self-body image is particularly amenable to intervention because it allows progressive, repeated, and low-threshold delivery of self-compassion and exposure-based practices, adapted over time in a stigma-sensitive manner. DTx can help initiate self-compassion and self-acceptance practices, both of which have established links to psychological well-being [106]. These interventions can take the form of guided meditation or gradual mirror exposure exercises. Regular use of self-affirmations and a focus on health-related rather than appearance-related changes can further support the development of self-compassion. Moreover, interventions should focus on health literacy, such as personal testimonials, educational videos on body diversity, or challenges related to recognizing digitally altered images in the media. Fear of judgment or social scrutiny can be addressed through peer support or interactive coaching on how to respond to potential comments. These elements can help DTx users gradually build social exposure in a progressive and supportive manner.

DTx platforms can personalize body-image interventions by tailoring exposure practices, self-compassion modules, and stigma-features (e.g., optional anonymity, moderated communities) to baseline distress and avoidance patterns. Key personalization include: exposure pathways with difficulty-adjusted practices according to body dissatisfaction levels and clinician notifications for possible dysmorphic disorder screening, self-compassion modules deliveries with self-affirmation writing exercises and cognitive restructuring of self-critical automatic thoughts using CBT thought records, and a stigma-safety architecture with optional anonymity in peer communities, moderated discussions and algorithmic contents filtering potentially triggering content. Safety protocols must address iatrogenic risks including: exposure exercises including acute psychological distress requiring clinician oversight with clear discontinuation criteria, and active moderation of peer communities. Evidence for digital delivery of exposure-based body image intervention remains limited, requiring controlled trials with psychological safety endpoints.

#### Social support

Social support is an important factor in reducing the risk of obesity and associated complications, as well as in ensuring the effectiveness of treatments. DTx are particularly well-suited to provide continuous and tailored social support. In fact, it can structure, moderate, and personalize peer interactions over time, while enhancing perceived support to sustain adherence and engagement, thus providing an optimal environment for peer support [107]. For users who are open to social support, DTx should integrate social network-like features, such as group challenges or anonymous discussion groups. Additionally, involving close relatives in the therapeutic process, for instance, by implementing shared support plans, could further enhance compliance and treatment success. However, DTx solutions that include discussion groups must ensure robust moderation to prevent the spread of misinformation and the circulation of potentially harmful comments.

DTx can implement graded social support integration based on user preferences and phenotype, with implementation requirements addressing both engagement optimization and safety organization. For phenotype-based pathways, users scoring low on social support access autonomous pathways with optional peer features presented as opt-in rather than default. Conversely, high social users receive interventions including facilitated peer support groups, family involvement modules with shared features, and frequent clinician messaging. However, privacy and safety infrastructure must include strong privacy controls, including explicit consent workflows for any data family sharing, pseudonymization in peer groups, and data governance policies specifying who accesses shared content. People should exit peer features at any time, without penalty or loss of core app functionality, with alternative solo pathways providing equivalent content through different modalities, thus preserving autonomous engagement options.

#### Health literacy

DTx may address health literacy because they can continuously assess users’ understanding and adapt the format, depth, and timing of information to support progressive knowledge integration. Then, health literacy levels should be regularly assessed. Depending on the results, recommendations should be adapted accordingly [108]. If literacy is low, content should be simplified and guided; conversely, if literacy is high, content can be more in-depth. Various formats for knowledge transmission can be considered, ranging from images and videos for lower literacy levels to advanced analytical content for those with higher literacy. Continuous monitoring of literacy levels will allow for progressive improvement in knowledge acquisition. These adaptations should also be aligned with the patient’s healthcare journey, ensuring that users better understand and integrate healthcare professionals’ recommendations.

DTx can tailor education to health literacy through adaptive content delivery strategies that assess comprehension rather than relying solely on demographics. Content adaptation must be multimodal: users with low-level literacy receive microlearning interventions, simplified language options, and enhanced visual aids; users with high literacy access technical content to satisfy information-seeking needs. Literacy levels are checked with embedded comprehension assessment, following educational modules, with brief teach-back checks framed as reflective exercises (multiple-choice responses). An integration with clinical care is necessary with automated generation of plain-language summaries following medical appointments, medication instruction clarification, provider notification of comprehension gaps, and pre-visit preparation tools, including question prompt lists. Equity considerations require testing across diverse populations inkling limited language proficiency users, older people, and users with learning disabilities, to ensure adaptive systems enhance rather than exacerbate health disparities.

## Recommendations and limits

The results of this narrative review suggest that integrating psychological phenotyping and management into obesity-focused DTx could improve patient engagement and, consequently, enhance therapeutic effectiveness. The objective was to present the literature on various psychological dimensions of obesity while providing recommendations for the development and implementation of DTx.

It is recommended to develop, adapt, or analyze psychological assessment and management tools according to these dimensions. Validated questionnaires can rapidly assess psychological dimensions including personality, motivation, and emotional regulation. However, their integration in DTx platforms requires psychometric validation in digital administration formats, demonstrated clinical utility, and algorithm transparency.

It is important to point out that for certain psychological factors, the use of questionnaires to make a clinical assessment is sometimes limited and does not allow for a precise and complete assessment that is why human intervention remains essential to bridge the gap in therapeutic guidance. Moreover, human contact with a healthcare professional is also essential for many people, which is why DTx is not intended to replace healthcare professionals but rather to support existing care pathway, this can be partially automated with personalized chatbots. DTx are intended to augment rather than replace healthcare professional expertise, serving as decision support tools and extending care between appointments rather than substituting for clinical evaluation.

Additionally, DTx can serve as remote monitoring tools. Personalized content must be tailored to different levels of health literacy, ensuring that users can fully engage with DTx. This psychological phenotyping and management should be adaptable over time, continuously providing relevant content to users.

The primary challenge of DTx lies in the scientific validation of the employed tools, which must meet high therapeutic standards. Adaptive algorithms should also undergo validation through longitudinal studies and integration into standard care protocols delivered by healthcare professionals, such as general practitioners. User attrition rates in obesity-focused DTx remain a major concern. Although content personalization is crucial, over-personalization should be avoided as it may reduce users’ autonomy, limit exposure to diverse content, and potentially increase cognitive overload. Further studies are needed to evaluate the actual utility and potential drawbacks of highly personalized approaches. Additionally, DTx should be accessible to all. It is essential to test these interventions among vulnerable populations (e.g., individuals experiencing financial insecurity, older adults, or those with disabilities) to ensure equitable access to care.

Finally, psychological phenotyping introduces specific risks beyond general DTx concerns. Automated assessment may miss atypical presentations. Personality-based tailoring may inadvertently limit users’ exposure to diverse coping strategies, potentially reducing their ability to adapt flexibly to different situations. Intensive psychological monitoring may induce hypervigilance in perfectionistic phenotypes or individuals with obsessive-compulsive traits, potentially worsening rather than improving psychological outcomes. Algorithmic bias in phenotyping tools may disproportionately misclassify underrepresented populations if training datasets over-represent certain demographics. These risks underscore the importance of clinician involvement in monitoring user responses and adjusting interventions accordingly, rather than relying solely on automated pathways. However, users must know who has access to these data and retain meaningful control over data-sharing decisions.

In conclusion, DTx represent a major advancement in obesity management by providing accessible, scalable, and personalized solutions. However, their effectiveness remains limited if they fail to account for the psychological complexity of patients. Integrating psychological phenotyping would enable DTx to go beyond traditional indicators (BMI, blood pressure) by offering targeted interventions tailored to users' personality, motivation, emotional regulation, and social support levels. Moreover, DTx must respond to specific requests: accessible, high-quality content, temporal strategies, gamification, and attractive design. This approach may enhance long-term engagement while reducing the high dropout rates often observed in traditional DTx programs.

However, several challenges must be addressed: ensuring the scientific validity of personalization tools, protecting patient data ethically, and striking the right balance between individualization and ease of use. Future research should explore the actual impact of psychological phenotyping and management on DTx effectiveness through randomized and longitudinal studies, assessing outcomes such as weight loss, treatment adherence, and psychological well-being. Recent neurobehavioral frameworks further support the relevance of phenotype-informed approaches for digital therapeutics in obesity, highlighting how mechanism-driven personalization may enhance long-term effectiveness [93].

Ultimately, incorporating psychological factors into DTx presents a major clinical and technological opportunity. It has the potential to revolutionize obesity management by introducing a more human-centered, adaptive, and effective approach, benefiting both patients and healthcare professionals, contingent on rigorous validation, appropriate clinical integration, and demonstrated superiority over existing care models.

## Author contributions

The authors’ responsibilities were as follows – CP: conceptualization, methodology, validation, final content, writing – original draft and review and editing; MG: methodology, validation, writing – review and editing; PD: supervision, validation; AH: supervision, validation, writing – review; and all authors: read and approved the final manuscript.

## Funding

No funding or support were obtained for this research.

## Conflict of interest

The authors report no conflicts of interest.
